# The size and behavior of virtual objects have influence on functional exercise and motivation of persons with multiple sclerosis: a randomized study

**DOI:** 10.1038/s41598-022-24046-3

**Published:** 2022-11-12

**Authors:** Imre Cikajlo, Alma Hukić, Anja Udovčić Pertot

**Affiliations:** 1 University Rehabilitation Institute Republic of Slovenia Soča, Linhartova 51, 1000 Ljubljana, Slovenia; 2 School of Engineering and Management, University of Nova Gorica, Vipavska 13, 5000 Nova Gorica, Slovenia

**Keywords:** Psychology, Medical research, Multiple sclerosis, Engineering

## Abstract

The consequences of multiple sclerosis are problems with limb movement, coordination, and vision. Heretofore a combination of therapy and additional medications can alter the course of the disease and reduce upper extremity disability. We developed a virtual environment for pick-and-place tasks as a supportive tool to address the problem of challenging task in occupational therapy. The primary objective of the study was to investigate the influence of size and bounce on proximal and fine motor performance and intrinsic motivation. The secondary objective was to examine how the absence of challenge may decrease intrinsic motivation and heart rate. The randomized trial involved 84/107 eligible inpatients with multiple sclerosis. They were divided into 4 groups by computer randomization: Group 1 small and bouncing, Group 2 small and non-bouncing, Group 3 large and bouncing, and Group 4 large and non-bouncing virtual cubes. Each participant completed 50 sessions of up to 2 min each in approximately 14 days. Before commencement of the study the participants completed visuospatial and cognitive tests. Participants' subjective experiences were assessed daily using the intrinsic motivation inventory. Before and after the study, the box and block test and the 9-hole peg test were administered. Kinematic analysis showed significant differences between groups (average manipulation time *p* = 0.008, inserted cubes *p* = 0.004). Group 4 was the most successful (inserted cubes > 9) and the fastest (63.4 SD 25.8 s), but had low pressure/tension and heart rate. Group 1 was the slowest (88.9 SD 28.2 s) but had increased interest/enjoyment in the task under higher pressure/tension. There were substantial differences in intrinsic motivation between the 1st and last sessions within groups (Cohen's U3 < 0.3 or > 0.8). The size and behavior of virtual objects may be important for training proximal movements and fine motor skills in people with multiple sclerosis. Furthermore, the demonstrated approach proved to be effective and may reduce upper extremity disability in the long term if intrinsic motivation can be sustained longer with a challenging task.

*Trial registration* The small scale randomized pilot trial has been registered at ClinicalTrials.gov Identifier: NCT04266444, 12/02/2020, https://clinicaltrials.gov/ct2/show/NCT04266444.

## Introduction

Multiple sclerosis (MS) is a demyelinating disease that affects the brain and spinal cord (central nervous system). The MS typically occurs in young adulthood, is chronic and progressive, and has a variable and unpredictable course^1^⁠. The disease is considered an autoimmune disorder that attacks the sheathing of nerve fibers (myelin) and causes dysfunction of the connections between the brain and spinal cord. The cause of MS is not fully understood, and the disease can lead to permanent disability. To date, there is no single pharmacological treatment to cure MS. Symptoms and quality of life can be alleviated with medications^2^⁠, which can influence the course of the disease to some extent. A complementary comprehensive multidisciplinary rehabilitation program establishes short- and long-term rehabilitation goals, rehabilitation plans, and interventions, and monitors and adjusts the goals and plans according to the needs of the patient and the stage of the disease^3^⁠. The impact of rehabilitation programs is usually assessed by Expanded Disability Status Scale (EDSS)^4^⁠, a method of quantifying disability in people with multiple sclerosis (PwMS) and monitoring changes in disability level over time, completed by a physician, neurologist, or specialist in physical and rehabilitation medicine.

Upper limb dysfunction is a very common consequence of MS and is often caused by tremor, sensory deficits, weakness, or loss of dexterity^5,6^⁠. Loss of motor skills affects activities of daily living (ADL), quality of life, and employment status^7,8^⁠. Clinicians and researchers have been actively developing neuromotor treatments to reduce the impact of upper limb disability, increase quality of life, and improve prognosis in PwMS. Neuromotor rehabilitation of PwMS includes assessment of manual dexterity, range of motion, strength, sensation, and coordination. Spooren et al^9^⁠ studied neuromotor training programs and reported improvement in arm and hand performance in PwMS. The authors emphasized the importance of individually tailored and progressive tasks for clinical progress. Reducing upper limb dysfunction is important for ADL^10^⁠, especially for proximal aspects and also for fine motor tasks. Virtual reality (VR) and exergaming allow task variability from a simple to a complex environment, customizable objects even in real time and have been suggested as potential aids^11^⁠, whether non-immersive or fully immersive devices^12^⁠. There is no evidence that such an approach is better in terms of clinical outcomes, but rather equivalent to the traditional approach. Studies have reported that VR and exergaming can effectively improve upper extremity motor function^13^⁠, may play an important role in ADL^14^⁠, and improve performance during high intensity exercise, likely due to increased enjoyment^15^⁠. Furthermore, VR and exergaming technology enable interventions related to cognition, mood, and ADL in combination with motor function^16^⁠. Additionally, assessment of movement, behavior, and progress tracking is possible, providing insights into mechanisms that validated clinical tests lack. With virtual objects, one can create individualized tasks for each PwMS and change object properties according to patients' visual, motor, or cognitive abilities, even in real time. Presumably, a challenging task can increase intrinsic motivation, often also pressure and tension. The higher pressure and tension may result in increased heart rate (HR), given the large body of literature on stress and anxiety increasing HR. Therefore, we designed a VR task that includes proximal movements and fine motor tasks to primarily test four different arrangements of virtual cubes; from challenging small and bouncing to easy-to-handle large and non-bouncing virtual cubes. The set-up allows occupational therapists to select the appropriate level of difficulty for individuals with disabilities based on cognitive, fine or gross motor skills to reduce the impact of disability caused by MS.

The aim of the randomized pilot study was to investigate the influence of the size of the virtual cubes and their bounce on proximal and fine motor performance and intrinsic motivation of people with disabilities. We hypothesized that PwMS may have difficulties challenging small virtual objects, resulting in higher heart rate and intrinsic motivation although the approach would be effective. In contrast, we expected that PwMS might find the task with large and non-bouncing virtual cubes easy and less challenging.

## Materials and methods

### Study design

A randomized pilot study with PwMS was designed. Participants were enrolled according to the inclusion and exclusion criteria (“Participants” section). Baseline clinical assessment was performed on the day participants were randomized to one of the four groups. All the PwMS participated in the post-treatment clinical assessment. Primary outcome measures were exergaming and kinematic outcomes, which provide information on the mechanisms of proximal movements and fine motor skills. Clinical testing and intrinsic motivation with additional heart rate information were considered secondary outcome measures.

### Rehabilitation system

**Figure 1:**
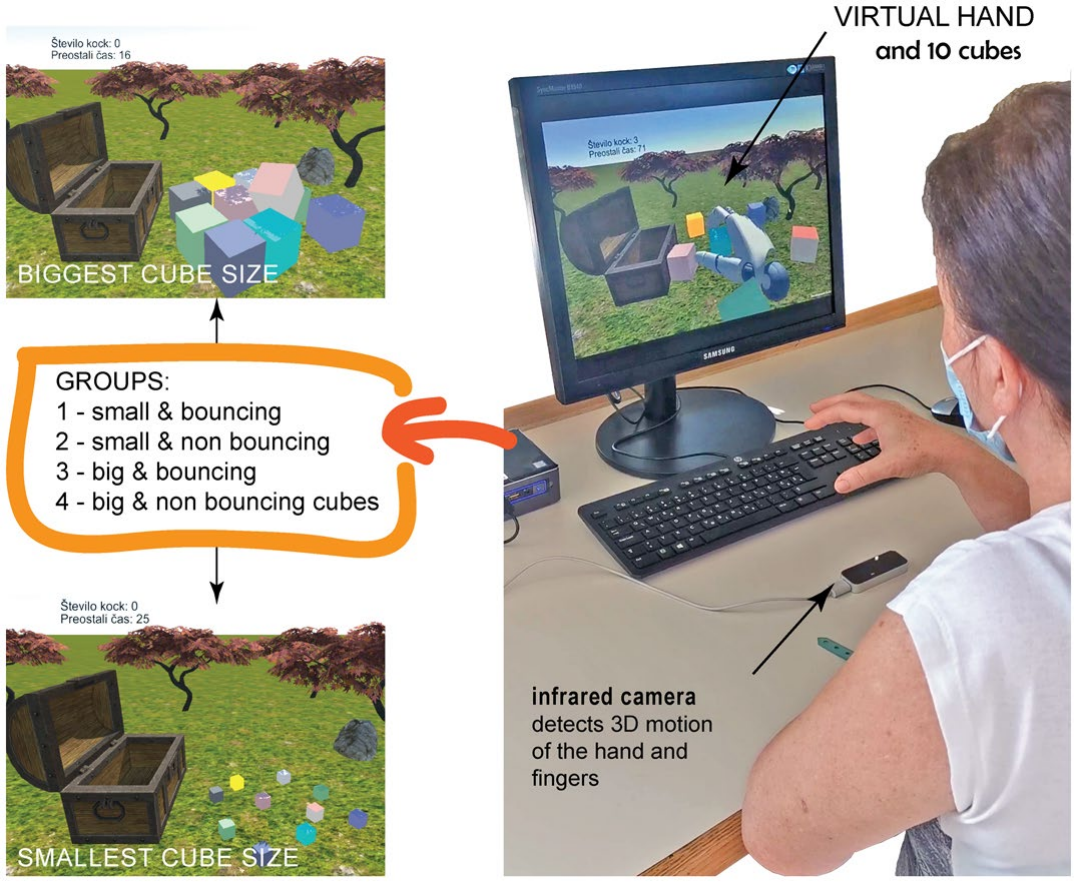
Exergaming setup. LMC detects hand and finger movements for interaction with the virtual environment. Small and large cubes with different bouncing characteristics were used to define 4 variants of the exergame.

The rehabilitation exergaming system is based on 3D kinematics captured by the Leap Motion Controller (LMC, Ultraleap Inc. Mountain View, CA, USA) infrared camera system with Orion SDK. The LMC is a small 80 × 30 mm optical tracking device with a 140 × 120 degree field of view and a tracking range between 10 and 60 cm. It detects hand and finger movements with sufficient accuracy^17^⁠ and transmits the data to the powerful computer with GPU via USB at 100 Hz. The robust and reliable skeleton model was modified in Unity3D (Unity Technologies, CA, USA) and used to track the kinematics of the hand and fingers. A virtual model of the hand combining the kinematics with a scenery in a virtual environment was designed and developed as a prototype of the exergame^12^⁠. The scenery consists of 10 virtual cubes of different colors but the same size and an open treasure chest in the green virtual field (Fig. 1). The goal of the exergame was to pick up all the cubes one by one with the virtual hand and place them in the open chest within two minutes. For the study, we determined 2 variable parameters: the size and the (non)bounce of the virtual cubes. The (non)bounciness of the virtual cube was adjusted in the virtual world by choosing the appropriate virtual weight and virtual surface made of plastic or rubber. The responsible researcher could choose from 4 different options that combined the size and the (non)bouncy surface of the cubes.

### Participants

**Figure 2:**
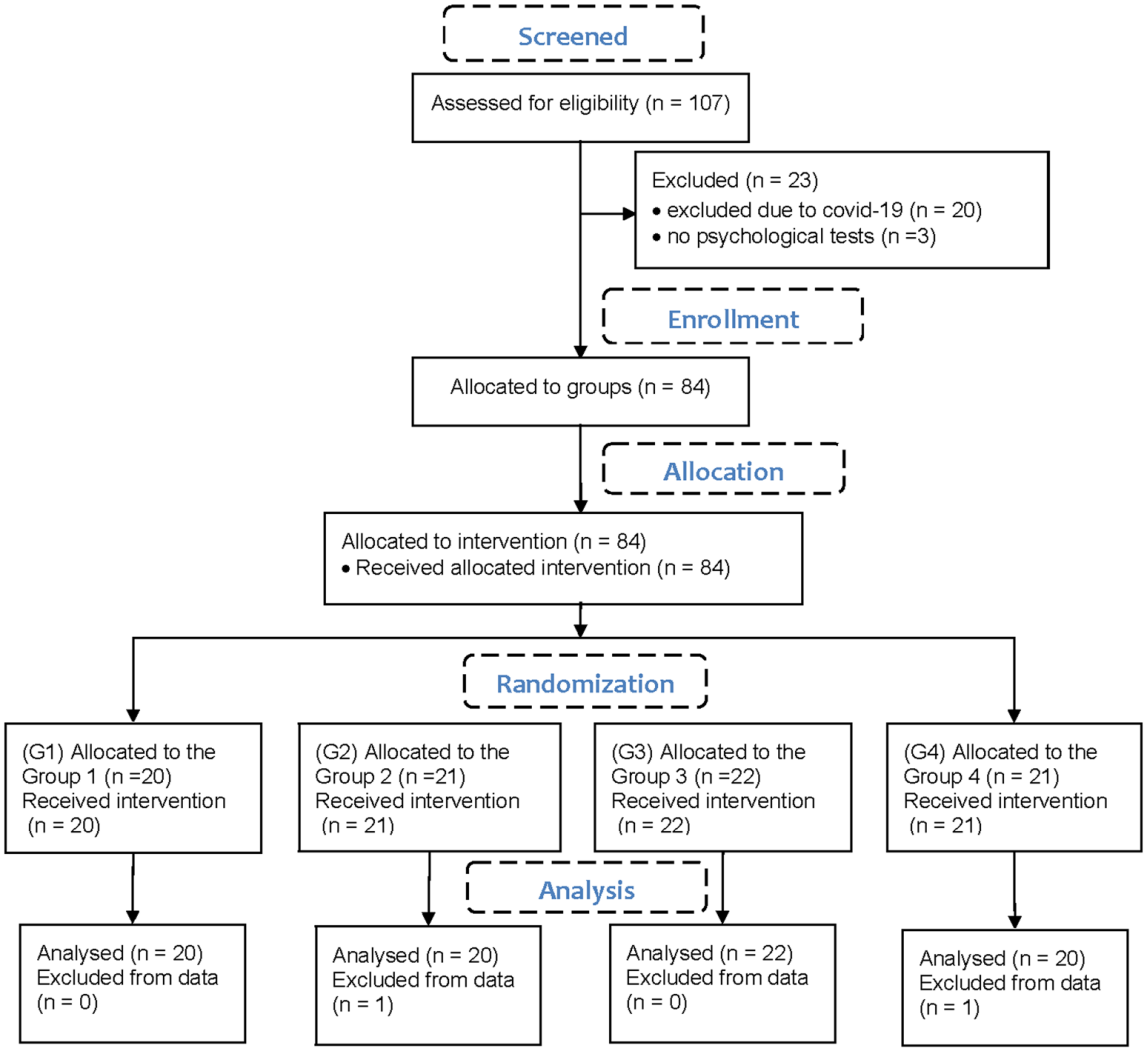
CONSORT flowchart.

In the randomized pilot study from February 2020 to September 2021, we recruited 107 local hospital inpatients diagnosed with multiple sclerosis (37 male, 70 female, age 51.1 ± 11.5 years) who were eligible for the study according to the inclusion criteria. Participants with relapsing–remitting, secondary progressive, and primary progressive multiple sclerosis^18^⁠ briefly underwent a Mini Mental State Examination (MMSE) to determine the severity of cognitive impairment with a maximum score of 30. Patients were also tested with the Expanded Disability Status Scale (EDSS)^4^⁠ disability assessment tool for PwMS to determine the degree of neurological impairment based on the degrees of functional systems and sensory systems (0.0 normal—9.5 highly disabled). The inclusion criteria were 1. no visual disorders (optic neuritis, diplopia, nystagmus, or scotoma), 2. preserved upper extremity function, 3. MMSE >  = 24. Twenty patients were excluded from the study because of the onset of covid-19. Three patients did not complete all required neuropsychological testing due to the absence of the neuropsychologist and were therefore excluded from the study. Eighty-four patients who were able to walk a short distance with a walker and use their arms and hands for activities of daily living (MMSE 27.6 ± 2.1, EDSS 5.8 ± 1.2) were enrolled in the study and assigned to the intervention in 4 groups (Fig. 2). Enrolled participants were divided into 4 groups by computer randomization to obtain equal numbers of participants per group. All participants received the same amount of therapy and followed the same study protocol but used the exergame with different types of virtual cubes:

- Group G1: small and bouncing cubes (N = 20)
- Group G2: small and non-bouncing cubes (N = 21)
- Group G3: big and bouncing cubes (N = 22)
- Group G4: big and non-bouncing cubes (N = 21).

The study was approved by local ethics committee and all participants voluntarily provided a written consent. Participants could withdraw anytime without providing a reason.

### Research protocol

Participants were seated in front of a table with a laptop. The camera was placed on a table in front of the participant to cover the optimal working area (Fig. 1). If needed, an additional support for the participant's arm would be provided. Participants were asked to take the virtual cubes with the more affected hand and place them one by one in the open treasure box (Fig. 1) within 120 s, enough time to finish the task successfully^12^⁠. The cube would disappear if it was correctly placed in the chest. If all 10 cubes had been successfully relocated before the time expired, the task would be considered completed. The exergame started with 10 cubes randomly distributed in the virtual environment. The virtual model of the cubes (size, (non) bouncing surface) was selectively determined for each group and was the same for all members of the group.

Each participant completed 50 training sessions, 5 per day for 10 days over two weeks. Each session lasted up to 2 min, and the total daily training, including instructions and short breaks between sessions, did not exceed one hour. All participants voluntarily completed the modified Intrinsic Motivation Inventory (IMI)^19^⁠ immediately after each session^12^⁠.

All participants completed the Box & Blocks Test (BBT)^20^⁠ and 9 Hole Peg Test (9HPT) functional tests before and after treatment. Visuospatial cognitive tests, Delis-Kaplan Executive Function System (D- KEFS)^21^⁠, Judgment of Line Orientation Test (JLO)^22,23^⁠, and Frontal Assessment Battery (FAB)^24^,⁠ were performed before treatment in the inpatient hospital.

### Data assessment

#### Neuropsychological assessments

The visuospatial cognitive tests, D- KEFS, JLO, and FAB were administered before the start of inpatient treatment by a clinical psychologist who was properly trained in test administration of these metrics. We used a scaled score for all subtests of D- KEFS: visual scanning, number sequencing, letter sequencing, number-letter alternation, and motor speed. The scale ranges from 0 to 19 with an average score of 10 points; a score of 16 and above would indicate very good performance, between 14 and 16 would indicate very good performance, between 12 and 14 would indicate average performance, 8–12 would indicate average performance, 6–8 would indicate low average performance, 4–6 would indicate borderline performance, and below 4 would indicate impaired/mild/moderate or severe performance (psychometric conversion table).

Our participants also completed the JLO purely visual test with 30 items. Each item is rated on a scale of 0–15: 0–5 severe deficit, 6–7 moderate deficit, 8–9 mild deficit, 10 borderline, and 11 + normal. For data presentation and statistical comparison between groups, we used percentile rank (psychometric conversion table): severe deficit (1), borderline (< 10), average (25–75), high average (75–90).

Finally, patients completed the cognitive test FAB, a brief cognitive and behavioral subtest^25^⁠. The literature reports the degree of cognitive impairment for the raw scores < 10 and mean (14.24) with standard deviation (3.43, N = 372) for the PwMS^26^⁠.

#### Kinematics of the hand

**Figure 3:**
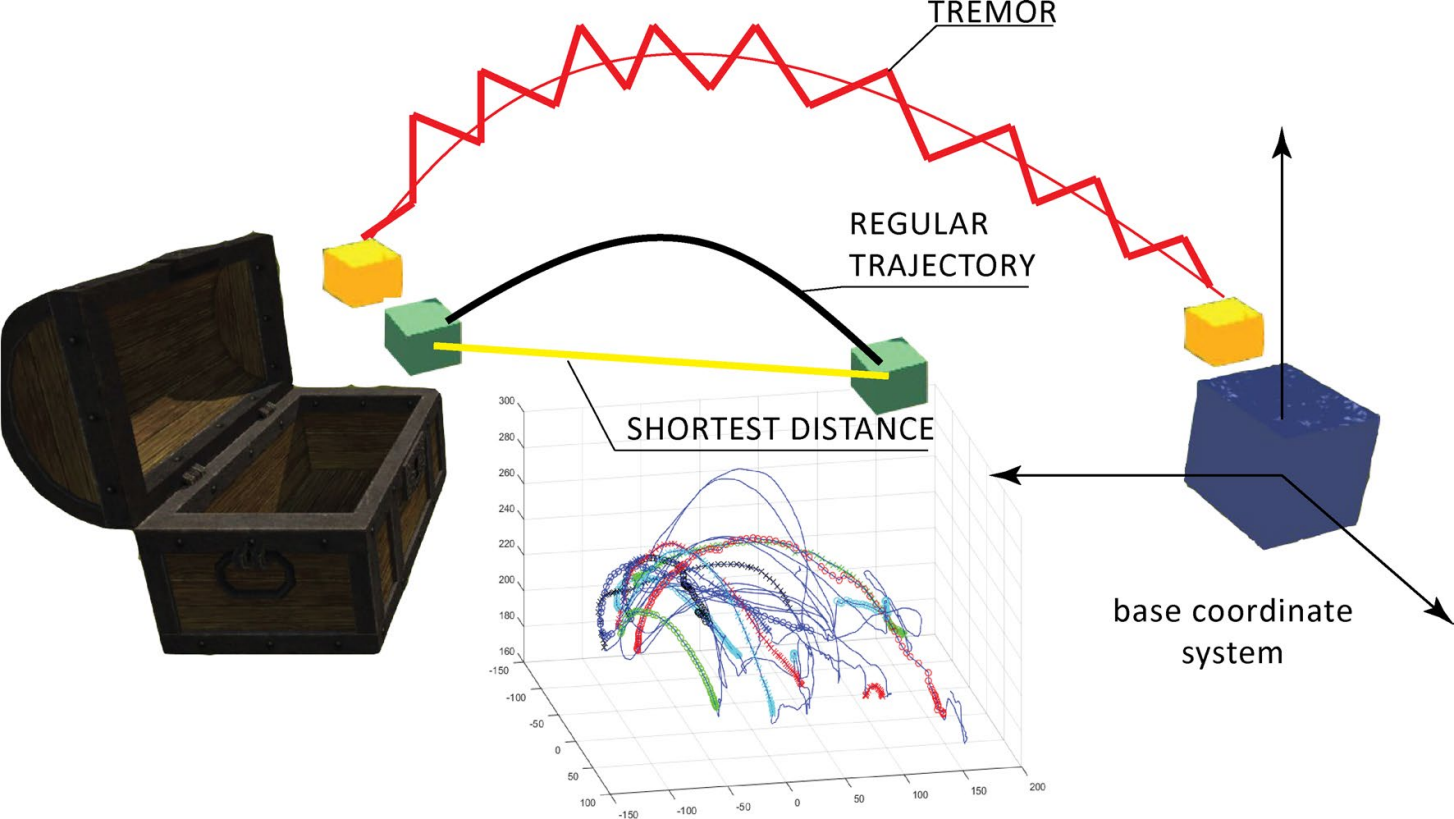
Tracking of the hand/palm with LMC provided trajectories in the base coordinate system that allowed calculation of the shortest distance, AToM, TNoT, and effective time of the exergame TfFTtE. The average tremor indicator (ATI) was determined from the jagged curve.

Objective parameters for the assessment of neuro-motor deficits in upper extremity exergaming have been developed^27^⁠ and used in patients with PD^12^⁠. The pose of the palm and fingers was assessed with the LMC and transformed into the base coordinate system. Hand kinematics (Fig. 3) and time were used to calculate the total time to manipulate all virtual cubes (TtoM), the average time to manipulate each virtual cube (AToM), the number of successfully inserted boxes (IN), the total number of trials (TNoT), the time from the first touch of the virtual cube to the end of the game (TfFTtE), the average shortest kinematic distance, and the average tremor indicator (ATI)^27^⁠.

The time from the first contact with a virtual cube until the last cube was placed in the chest was considered as the total time of the trial, unless the user did not complete the task. In the present study, we focused on the main indicators of time and score, i.e., AToM, TfFTtE, IN, and TNoT. We also report the ATI, since it is likely to be much larger for smaller cubes.

#### *Clinical functional test, intrinsic motivation inventory and heart rate*

The BBT and 9HPT were performed by a trained occupational therapist before and after exergaming sessions at the rehabilitation center. The BBT was the occupational therapist's tool to assess unilateral gross manual dexterity. The goal was to place the wooden cubes from one compartment to another within 60 s. The score corresponded to the number of cubes placed. The 9HPT is a standardized upper extremity test. The task was to pick up the nine pegs one at a time, place them in a hole, and then remove them. The total time was recorded.

Participants rated their experience with the exergame immediately after the daily sessions by rating the 8 statements of the modified^28^⁠ IMI^19^⁠ on a 7-point Likert scale. A score of 1 indicated complete disagreement and a score of 7 indicated full agreement with the particular statement. The statements were afterward classified into 4 categories:

Interest/Enjoyment

Statement 3: The game seemed very interesting to me.
Statement 7: Playing was fun.

Effort/Importance

Statement 1: I have made a lot of efforts to play the game.
Statement 4: I did my best.

Perceived Competence

Statement 2: I think it's a good game for me.
Statement 6: I am satisfied with my result.

Pressure/Tension

Statement 5: During the playing I was very tense.
Statement 8: During the game I felt under pressure.

Each category presented the IMI measure scale ranging between 2 and 14 points.

During the exergaming sessions, heart rate was constantly monitored on the participant's wrist using a smart wristband (Samsung Galaxy Fit-e, Samsung Inc. Korea).

### Data analysis

Matlab (MathWorks, Natick MA, USA) was used to extract raw kinematic data from the LMC data stream. We calculated the position of the palm and used it to calculate the motions in time and space. Mean values were calculated for all kinematic parameters for each group and sorted by chronological sessions. In addition, we performed the same analysis for the manipulation of the virtual cubes. A 4th order polynomial curve fit with normalization was used for plotting. Because the data were unbalanced, the equivalent of the Friedman test, the non-parametric Mac-Skilling test, was used^29^⁠. The significance level was set at *p* = 0.05.

Matlab Statistical Toolbox was used to calculate the mean, standard deviation, Levene's test, and analysis of variance (ANOVA) to examine the differences between groups. Kruskal–Wallis one-way analysis of variance reported group effects in psychological tests, and Friedman's non-parametric 2-way test for unbalanced data was used to test the hypothesis of group effect in BBT, 9HPT, HR, and IMI. The significance level was set at *p* = 0.05.

Cohen's U3 index^30^⁠ was used to find effect sizes in BBT, 9HPT, IMI, kinematic and exergame parameters. The U3 index defines the proportion of data from a given group that were smaller than the median values of the other group. There would be no effect at U3 = 0.5 and maximum at 0 if all the group's data were above the median of the other group, or 1 if all the group's data were below the median of the comparison group (effect size: small 0.4/0.6, medium 0.3/0.7, and large 0.2/0.8). Matlab Statistical Toolbox (MathWorks, Natick MA, USA) with Measures of Effect Size (MES) Toolbox^31^⁠ and GNU PSPP (Free Software Foundation, Inc., Boston MA, USA) were used for analysis.

### Ethics approval and consent to participate

The study (Approval Number: URIS202001) was approved by ethics committee of University Rehabilitation Institute, Republic of Slovenia and all participants provided an informed written consent. The procedure was in accordance with the principles of the Declaration of Helsinki on biomedical research on human beings, the provisions of Council of Europe Convention on the Protection of Human Rights and Dignity of the Human Being with regard to the Application of Biology and Medicine (Oviedo Convention) and the principles of Slovenian Code of medical ethics. The authors confirm that all ongoing and related trials for this drug/intervention are registered (ClinicalTrials.gov Identifier: NCT04266444, 12/02/2020, https://clinicaltrials.gov/ct2/show/NCT04266444).

## Results

### Differences between the groups

2 participants (1 from group G2 and 1 from group G4) did not complete all daily sessions. Therefore, a total of 82 participants' data were used for calculation. No significant statistical differences were found between the participating groups in age, sex, and EDSS with Levene's test and ANOVA, but for the MMSE (Table 1), the Kruskal–Wallis non-parametric test showed significant differences between groups 1 and 4.

**Table 1:** Mean differences between the groups.

Variable	Group (mean/SD)				Levene’s Test	ANOVA/Kruskal walilis Test
	G1	G2	G3	G4	(F/*p*)	(*p*-value)
Gender (M/F)	5/15	8/12	5/17	5/15	1.64/0.187	0.604
Age (mean/SD)	53.8/10.8	53.6/11.5	48.6/12.8	49.1/11.8	0.18/0.907	0.350
EDSS	5.8/1.2	6.0/1.2	6.1/0.8	5.6/1.4	1.56/0.206	0.550
MMSE	26.4/2.6	27.6/2.9	27.8/1.4	28.5/1.2	3.95/0.012*	0.056*

*Statistically significant differences (*p* < 0.05).

### Neuropsychological differences between the groups

**Figure 4:**
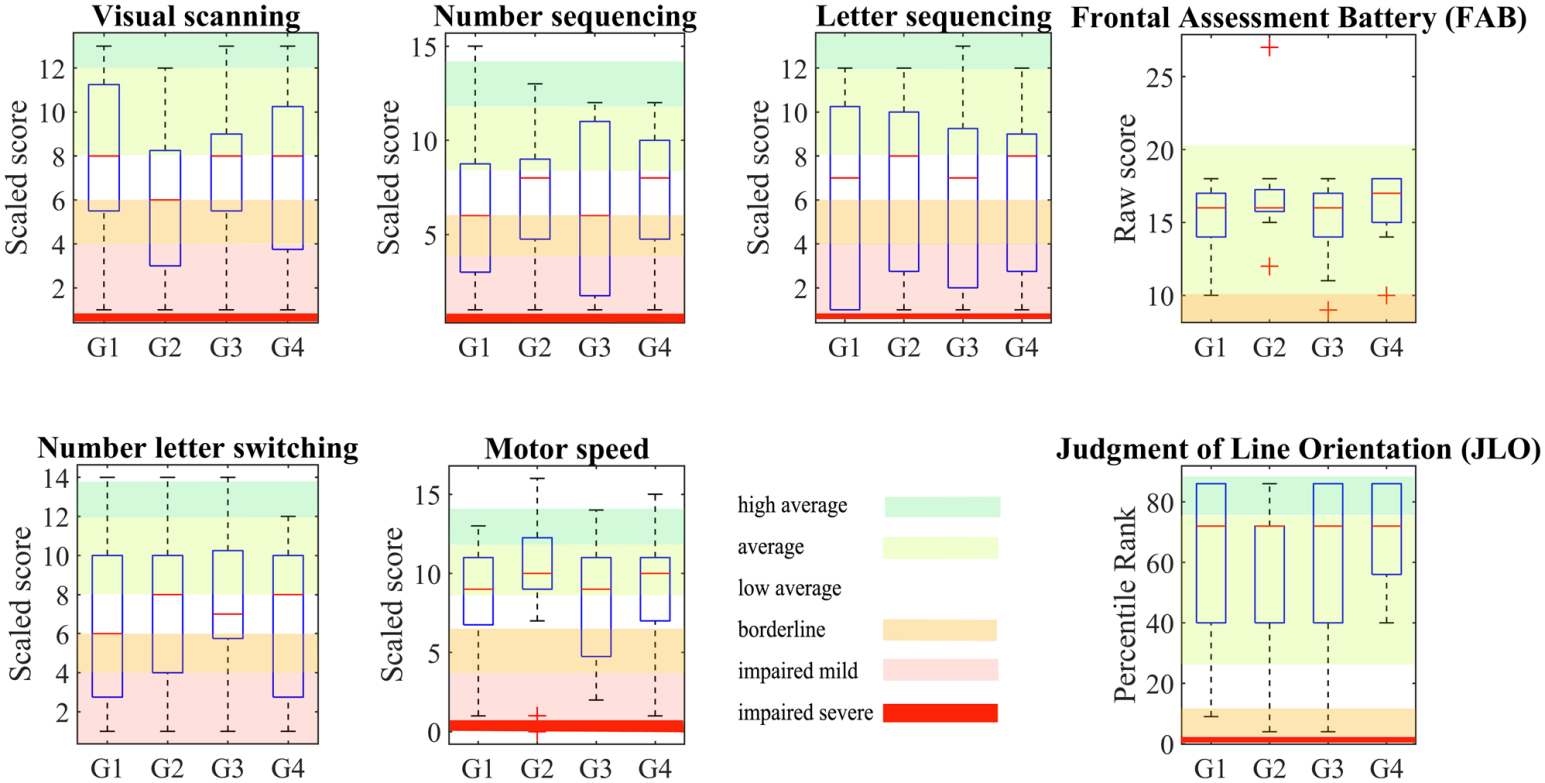
The cognitive tests D- KEFS, JLO, FAB showed rather small differences of median scores between groups and low average to high average scores, which means that the participants were not severely cognitively impaired.

The standardized test of higher-level cognitive functions, the Delis-Kaplan Executive Function System (D- KEFS), showed average (8–12 scaled scores) to low-average (5–7 scaled scores) performance on visual scanning, number sequencing, average on letter sequencing, and switching between numbers and letters, and average to high-average (12 + scaled scores) performance on motor speed functions (Fig. 4). Statistically, no significant differences were found between groups on all subtests of D- KEFS (*p* = 0.3376, *p* = 0.743, *p* = 0.9887, *p* = 0.9083, and *p* = 0.4365, respectively). All participants in all groups were cognitively unimpaired and had a FAB greater than 10 (15.9/2.3)^26^⁠. No statistically significant differences were found between groups (*p* = 0.1205). The visuospatial perception (JLO) measure has an average (25–75) to high average (75–90) rank (mean 62.1/26.2), also with no statistically significant differences between groups (*p* = 0.3813).

### Kinematics of the hand at exergaming

**Figure 5:**
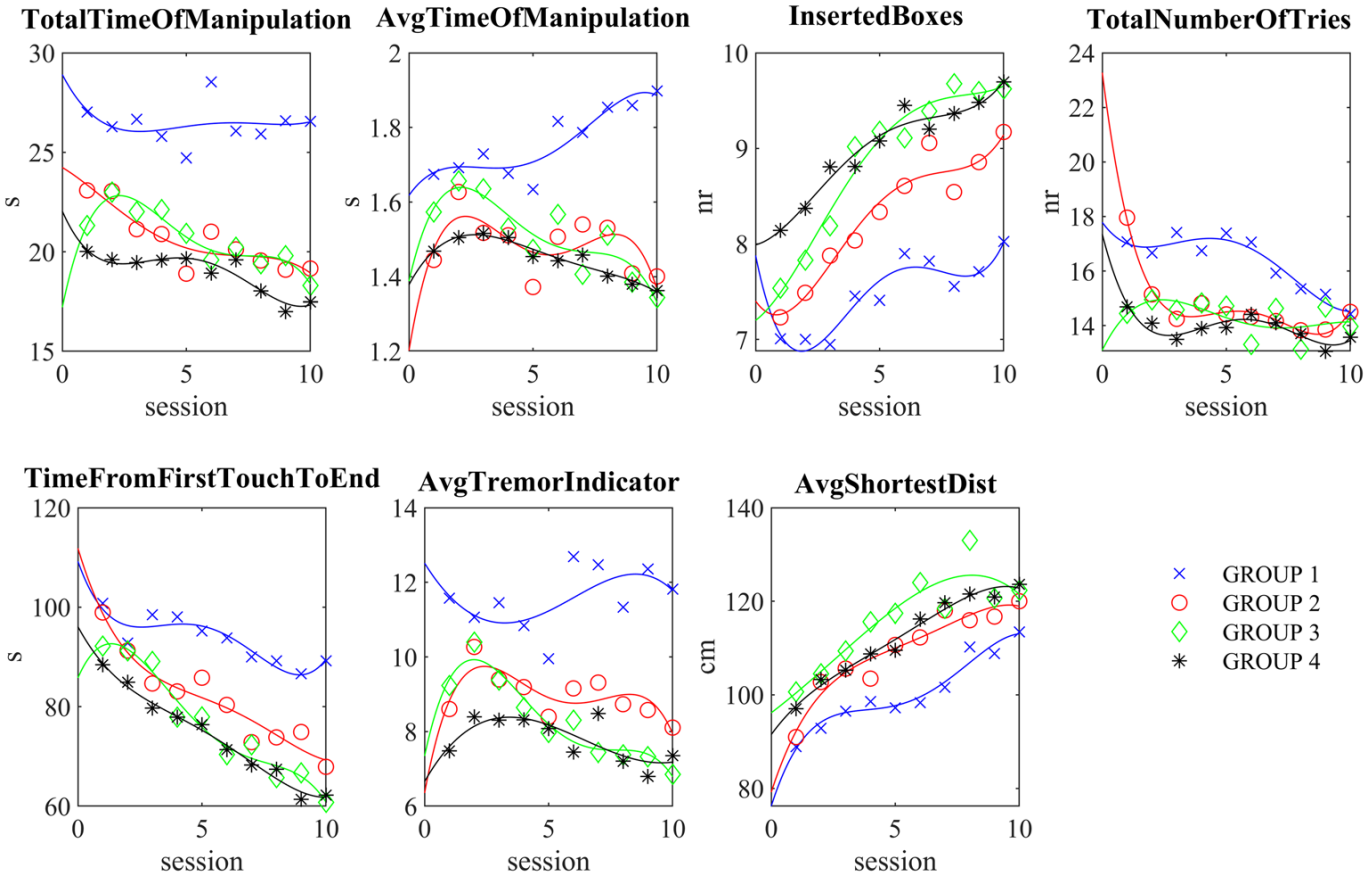
Kinematic and exergame parameters showed that group 1 (the most difficult task with small bouncing cubes) needed more time to finish the task and had less success (lower number of inserted boxes). In contrast, members of Group 4 learned to use the larger cubes more quickly and completed the exergames with all 10 cubes inserted after 10 sessions.

Participants in group 1 had the most difficult task with small and bouncing cubes, therefore the longest total time of manipulation TtoM and TfFTtE were substantially higher, up to 25%. Moreover, AToM indicated a longer time, especially at the end of the sessions, when participants paid attention to accuracy and success in exergaming. Indeed, all groups were more successful, with participants able to use more cubes and reduce TNoT. The average shortest distance increased fairly consistently and appropriately for all groups. Group 4 members succeeded in reducing manipulation time and TfFTtE, followed by a higher success rate in inserting cubes in a minimal number of trials. The ATI was greater when manipulating small objects (Fig. 5).

**Figure 6:**
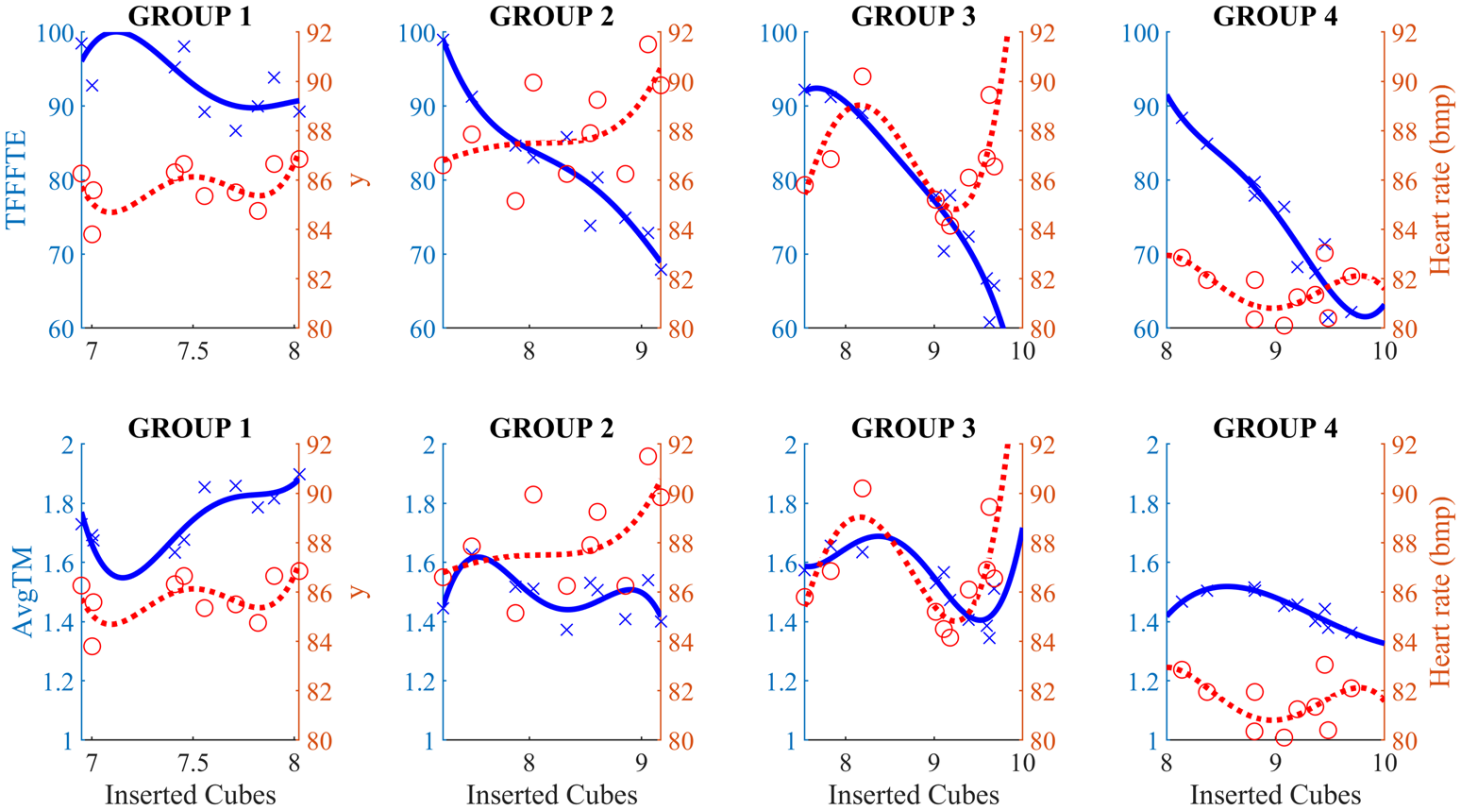
TfFTtE was substantially lower only in the groups (3, 4) that managed to complete the task with 9 or even 10 cubes inserted. However, it was obviously not easy for group 3 with much higher HR than group 4. The AToM was again higher in group 1 and shorter in group 4 with low HR.

Participants in group 1 barely managed to throw in an average of 8 cubes in 120 s, with AToM ranging from 1.7 to 1.9 s and HR remaining at 85.7 SD 0.9 bpm. TfFTtE was highest among all groups, averaging 100 s with 7 cubes inserted and 89 s with 8 cubes inserted (Fig. 6). Participants in group 4 were successful at exergaming, placing all 10 cubes and significantly reducing TfFTtE from 87 s (8 cubes) to 63 s (10 cubes) at low HR at 81.5 SD 1.0 bpm. Also, their AToM was the lowest among the groups.

Participants in groups 2 and 3 decreased TfFTtE from 98 to 67 s and 93 s to 63 s, respectively, to achieve a higher exergaming score. However, with a higher HR, 88.1 SD 2.0 and 86.6 SD 1.9, respectively. AToM was lower than in group 1 but higher than in group 4. Participants in group 3 were on average more successful at exergaming than group 2 (9.6 vs. 9.1 cubes). Statistically significant differences (*p* < 0.05) between groups in time-related actions, exergaming performance, and tremor index were found between the first and last session (Table 2).

**Table 2:** The mean outcomes of the TfFTtE (Time from first touch to the end), AToM (Average time of manipulation), IN (Inserted boxes), TNoT (Total number of tries) and ATI (Average tremor indicator) at 1st and last session.

	Group G1				Cohen’s U3	Group G2				Cohen’s U3	Group G3				Cohen’s U3	GroupG4				Cohen’s U3	Mac-Skilling
	1st		10th		U3	1st		10th		U3	1st		10th		U3	1st		10th		U3	*p*
	Mean	SD	Mean	SD	[CI]	Mean	SD	Mean	SD	[CI]	Mean	SD	Mean	SD	[CI]	Mean	SD	Mean	SD	[CI]	
TfFTtE	100.4	20.9	88.9	28.2	0.3[0.05–0.7]	98.9	15.1	67.9	27.5	0.0 [0.0–0.15]	93.8	24.1	63.6	23.3	0.15 [0.0–0.3]	87.5	22.6	63.4	25.8	0.14 [0–0.4]	0.0027*
AToM	1.72	0.82	1.90	0.47	0.6 [0.5–0.9]	1.44	0.55	1.40	0.45	0.45 [0.2–0.8]	1.59	0.67	1.35	0.35	0.4 [0.2–0.7]	1.43	0.58	1.34	0.49	0.5 [0.1–0.8]	0.0081*
IB	7.05	2.24	7.95	2.33	0.76 [0.4–0.9]	7.23	1.99	9.17	1.35	0.95 [0.8–1.0]	7.41	2.35	9.57	0.67	0.9 [0.5–0.9]	8.08	2.51	9.68	0.83	0.7 [0.5–0.9]	0.0038*
TNoT	17.0	5.56	14.1	1.77	0.43 [0.2–0.6]	17.9	6.51	14.5	4.2	0.15 [0.0–0.6]	14.6	3.49	14.3	3.80	0.35 [0.15–0.8]	14.5	5.40	13.72	3.93	0.2[0.05–0.6]	0.1308
ATI	11.9	7.06	11.9	3.89	0.7 [0.3–0.9]	8.60	3.05	8.10	2.86	0.6 [0.15–0.6]	9.76	4.71	7.38	2.31	0.35 [0.15–0.6]	7.41	1.77	7.38	3.46	0.2[0.05–0.8]	3.3922e- 05*

The Mac-Skilling non-parametric test was used to test the statistical differences (group x time, *p* < 0.05). Cohen’s U3 demonstrated the size effect between 1st and 10th session.

*Statistically significant differences (*p* < 0.05).

Additionally, we found differences in the TfFTtE and the IB between the assessments in the 1st session and the last, 10th session, substantially significant for all the groups (Table 2).

### Clinical outcomes and intrinsic motivation

The mean BBT increased from 48.45 SD 8.98 to 50.75 SD 9.86 cubes in the G1 group, 42.64 SD 11.47 to 47.18 SD 11.41 cubes in the G2 group, 47.50 SD 7.93 to 52.04 SD 9.28 cubes in the G3 group, and 41.38 SD 14.60 to 47.45 SD 13.76 cubes for the affected hand in the G4 group after the sessions. The outcomes of 9HPT demonstrated improvement in all groups (G4: 36.02 SD 17.22 vs 32.24 SD 20.24, G3: 28.39 SD 5.62 vs 26.29 SD 5.69 and G2 45.09 SD 26.10 vs 35.75 SD 22.28) except G1 (32.57 SD 16.31 vs 32.59 SD 33.50) with high standard deviations.

**Figure 7:**
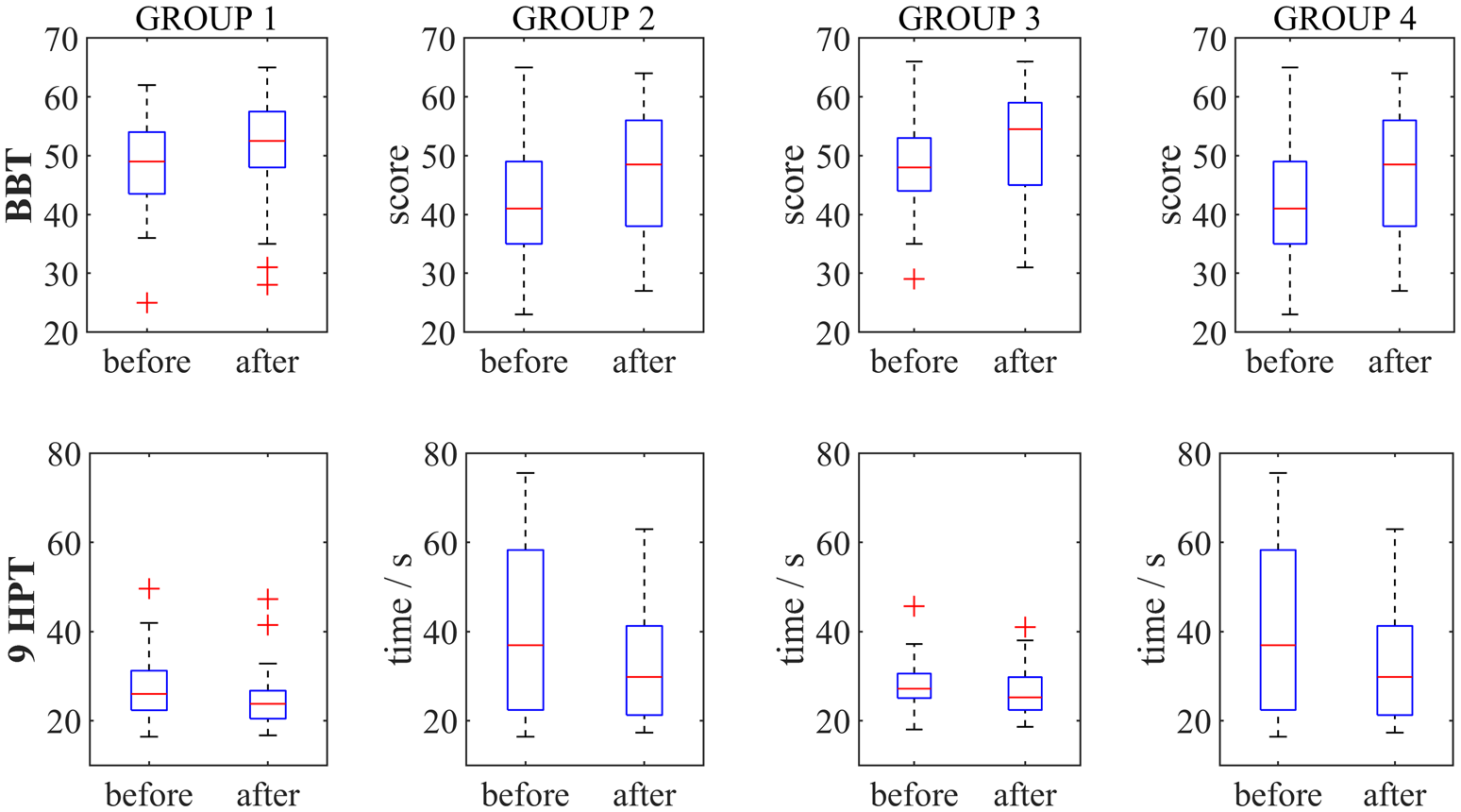
All participants improved their BBT and 9HPT performance (Cohen's U3), but no statistically significant differences were found between groups (BBT: $\upchi ^{{2}}$ = 4.099, *p* = 0.2509, 9HPT: $\upchi ^{{2}}$ = 4.480, *p* = 0.2142).

Effect sizes show substantial differences and are given with Cohen's U3 and 95% confidence interval (CI) for BBT U3 = 0.7 CI [0.35–0.9], U3 = 0.7 CI [0.4–0.9], U3 = 0.8 CI [0.5–0.98], and U3 = 0.6 CI [0.33–0.85] and 9HPT U3 = 0.4 CI [0.1–0.7], U3 = 0.4 CI [0.1–0.6], U3 = 0.2 CI [0.4–0.5], and U3 = 0.3 CI [0.5–0.6] for the G1, G2, G3, and G4 groups, respectively. Levene's test for homogeneity of variances rejected the hypothesis (3.41, *p* = 0.022), and the Friedman two-way test confirmed no statistically significant differences between groups (BBT: $\upchi ^{{2}}$ = 4.099, *p* = 0.2509, 9HPT: $\upchi ^{{2}}$ = 4.480, *p* = 0.2142). The (Fig. 7) shows the median values, 25th and 75th percentiles, and whiskers that are 1.5 times the interquartile range for BBT and 9HPT.

All participating groups showed significant differences between the first and last sessions (Table 3); for the "interest/enjoyment" and "perceived competence" measurement scales of IMI. However, the differences between groups were not statistically significant (*p* = 0.978). All groups except G1 demonstrated substantial differences (U3) for the "effort/importance and pressure/tension" measurement scales. Group G1 showed negligible changes in the scales "effort/importance" and "pressure/tension" (U3 = 0.4).

**Table 3:** The Cohen’s U3 coefficient demonstrated substantial differences in effect size between 1st and last session for IMI measure scales.

	Group G1	Group G2	Group G3	Group G4	Friedman
	Cohen's U3 [CI]	Cohen's U3 [CI]	Cohen's U3 [CI]	Cohen's U3 [CI]	
	Session 1 vs 10	Session 1 vs 10	Session 1 vs 10	Session 1 vs 10	p*
Interest/enjoyment	0.3 [0.1–0.8]	0.2 [0.0–0.8]	0.8 [0.3–0.9]	0.8 [0.15–0.9]	0.978
Effort/importance	0.4 [0.13–0.85]	0.2 [0.0–0.75]	0.9 [0.35–0.95]	0.7 [0.35–0.85]	0.216
Perceived competence	0.6 [0.4–0.9]	0.8 [0.5–1.0]	0.9 [0.68–1.0]	0.8 [0.38–1.0]	0.860
Pressure/tension	0.4 [0.1–0.7]	0.2 [0.0–0.65]	0.1 [0.05–0.45]	0.1 [0.0–0.5]	0.297

*Statistically significant differences (*p* < 0.05).

**Figure 8:**
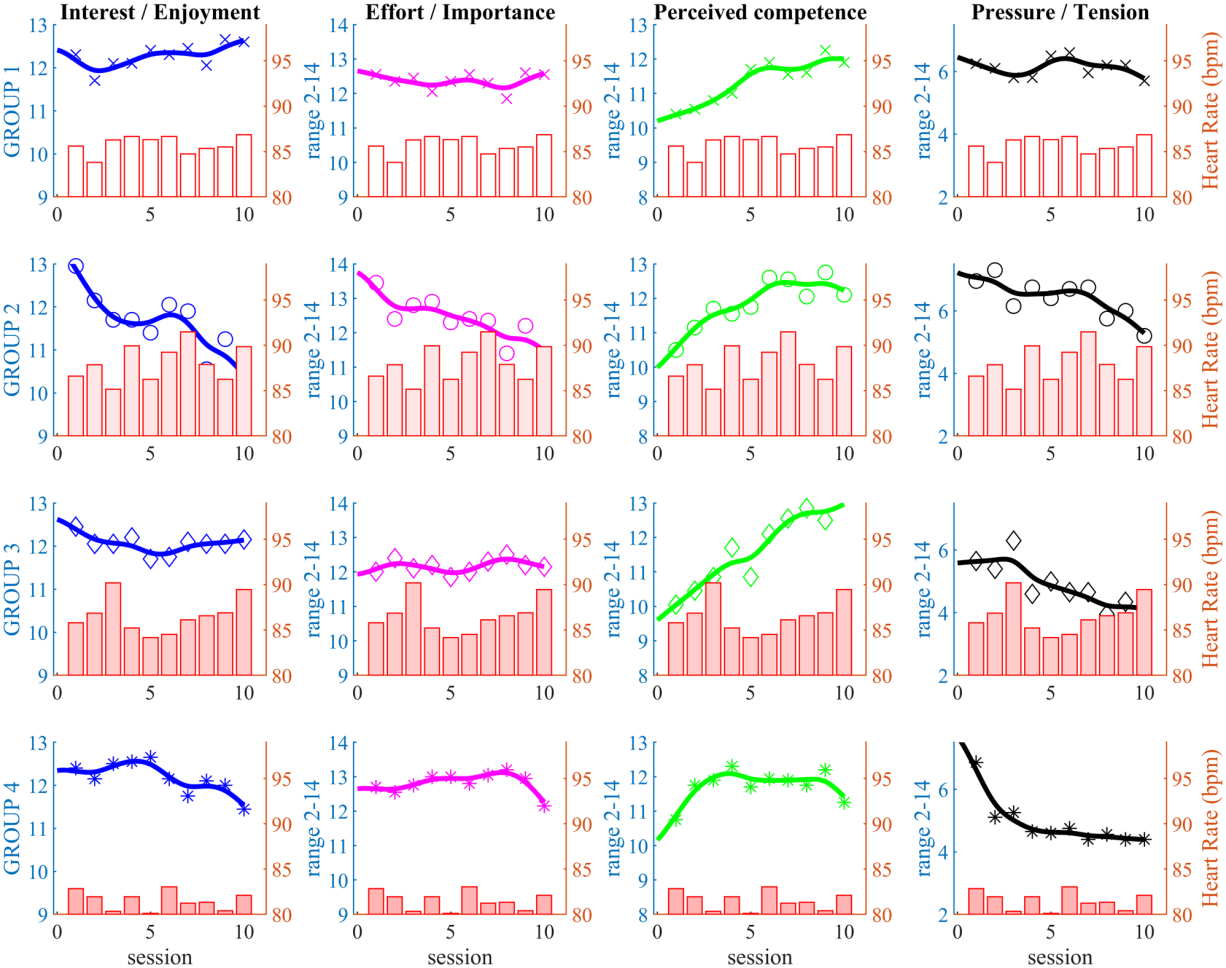
Group 1 participants were very interested in the exergame and maintained high levels of perceived competence, intensity and effort at the expense of constant pressure/tension and HR higher than at rest. In contrast, participants in Group 4 lost enjoyment due to the simplicity of the task. Also, at significantly lower HR than other groups, much less pressure/tension was required to successfully complete the task.

Group G4 had a consistently and statistically significantly ($\upchi ^{{2}}$ = 7.798, *p* = 0.050) lower HR during the exergaming session than the other groups (81.5 SD 1.0 bpm). The HR was highest on average in group G2 (88.1 SD 2.0 bpm), which also experienced the highest pressure/tension (Fig. 8). Pressure/tension was quite high throughout the sessions in G1, averaging HR 85.8 SD 0.9 bpm. The average resting rate HR of all groups was 78.5 SD 1.2 bpm.

## Discussion

### Exergaming performance and functional outcomes

In the study conducted with PwMS, groups were almost evenly randomized, with no statistically significant differences in gender, age, and EDSS. There were statistically significant differences between groups in visuospatial or cognitive abilities, but no patient was below the required MMSE score. The visuospatial test JLO and the cognitive and brief behavioral test FAB, administered before the start of the sessions, showed no critical or severe impairments, but rather average to low average scores according to age and educational norms^25^⁠, indicating that exergaming should not be a burden. Moreover, exergaming has been shown to be effective in people with Parkinson's disease^32^⁠, and a systematic review^33^⁠ also reported comparable results of exergaming and virtual reality-assisted balance and gait rehabilitation with conventional training. In addition, we hypothesize that practicing proximal movements and fine motor activities is important for brain excitability^34^⁠. Exergaming proximal movements and fine motor activities in the conducted study contributed to the significant changes in kinematic parameters and exergame scores in all groups. Participants obviously became familiar with the exergame and decreased the TNoT, which resulted in better scores in the IB. They also required less time for the pick-and-place task, which decreased AToM and also TfFTtE. However, this was not the case for group 1. The statistical test also showed significant differences between the groups. Group 1 required more manipulation time (AToM) for each virtual cube. Participants in this group experienced HR above rest, but they showed a high level of interest and enjoyment and kept the excitement high until the end of the training. Thus, the task with the small cubes with bouncing characteristics was confirmed as the most challenging task. In contrast, Group 4 mastered the task with the large and non-bouncing cubes in the first week and finished each session faster and quicker. They reported low pressure/tension and also the HR was much lower than the other groups. In the second week, their interest and enjoyment decreased, confirming our hypothesis that group members found the setup rather easy and less challenging. Most of these results are consistent with recent studies^35^⁠ reporting the effects of cognitive load on motor performance in people with disabilities. The study indicated a strong correlation of the results with the Montreal Cognitive Assessment—MoCA^36^⁠. The aforementioned study is also consistent with our findings that PwMS have a strong ability to improve motor control. We reported improvements in proximal movements and fine motor skills assessed with the BBT and the 9HPT. However, the differences in clinical tests between groups were not obvious, and it is difficult to determine which specific play parameter would be more clinically relevant. Nevertheless, improvement in kinematic parameters may provide insight into neuromotor mechanism^37^⁠ and is a good complement to validated clinical tests.

### Suggested exergaming parameters for higher motivation

The improvement in proximal movements and fine motor skills demonstrated by kinematic assessment can be valuable information for researchers, but clinicians value clinical studies with validated tests more. Kinematic analysis can shed light on clinical changes from a different perspective and reveal differences in the course of treatment that the clinical instrument often neglects due to its sensitivity^38^⁠. In this study, the substantial changes (Cohen's U3) in the intrinsic motivation inventory (IMI) can be explained by the kinematic analysis and the analysis of the play scores, especially in the interest/enjoyment and pressure/tension categories. The clinical tests showed an equal improvement for all groups, but the IMI reported a decrease in motivation and tension in all groups except group 1. Participants in group 1 handled small and bouncing virtual cubes that were difficult to grasp or pinch and often fell out of their hands. Members of Groups 4 and 3 did not have this problem; the latter only struggled with the bounciness of the virtual cubes. However, the bounciness was not negligible. We found the non-bounciness in group 2 less challenging (interest/enjoyment), but dealing with small virtual objects resulted in maintaining pressure/tension and the higher HR. According to our findings, we may rank the difficulty level from the easiest (group 4) to the most challenging (group 1). Such exergame parameters can be an important option to control participant engagement and maintain participant motivation. We have already shown promising results using the intrinsic motivation inventory as a key factor to motivate participants^39^⁠. Therefore, identifying optimal game parameters would be important to achieve medium-term motivation for telerehabilitation and home exercises.

### Limitations of the study and future work

The LMC has been validated with gold-standard optical measurement systems^40^⁠ and provides clinically acceptable and meaningful results^41^⁠, so our kinematic results can be considered reliable. However, we have found that the virtual hand occasionally rotates unexpectedly-an unpleasant event that significantly prolongs the task of picking up and putting down. When such an event occurred at least twice in a single session, we restarted and repeated the session. In case of muscle weakness, we could use passive or active dynamic arm support^42^⁠.

Changes in the HR were small for an average 50 year old individual which was expected given that the exergame was motor rehabilitation rather than exercise-based.

Despite the large number of participants, they were randomly divided into four groups, leaving room for a more extensive study. In the future, we could recruit more participants and gain solid knowledge about the correct choice of virtual object size, behavior or other virtual parameters in specific neuromuscular diseases or disorders.

## Conclusion

We have shown that the size and behavior of virtual objects have a significant impact on functional exercises and motivation of people with disabilities. The difficulty level of the task can help the patient to focus on the exergame, increase interest and effort, but consequently increase tension/pressure. Our expectations that dealing with small virtual objects would be challenging were confirmed. It is possible that bouncing played a key role, as tension in the group with large and non-bouncing virtual cubes decreased in less than a week and participants found the game easy and less challenging. The results could provide insight into kinematic strategies when using exergaming in occupational therapy. Moreover, developers might appreciate such information in designing the complex task for extensive and long-term use of such applications in rehabilitation medicine, especially in telerehabilitation.

## Data Availability

Anonymized data generated or analyzed during this study are available on request (contact: prim. Tatjana Erjavec, MD, tatjana.erjavec@ir-rs.si, University rehabilitation institute Republic of Slovenia Soča).
